# Does Gut-Endometrial Immunomodulation Promote Pregnancy in IVF Patients with Recurrent Implantation Failure and Chronic Endometritis Following *Escherichia coli* Nissle 1917?

**DOI:** 10.3390/jcm15156045

**Published:** 2026-08-03

**Authors:** Flora Caruso, Alfonso Manzi, Luigi Vigilante, Ida Strina, Alessandra Gallo, Attilio Di Spiezio Sardo, Maria Rosaria Fantuz, Giovanni Savarese

**Affiliations:** 1 Department of Public Health, School of Medicine, University of Naples “Federico II”, 80131 Naples, Italy; flora.caruso@hotmail.it (F.C.); lui.vigilante@gmail.com (L.V.); ida.strina@unina.it (I.S.); alessandra_gallo@hotmail.it (A.G.); attiliodispiezio@libero.it (A.D.S.S.); 2 Ames Centro Polidiagnostico Strumentale, 80013 Naples, Italy; rosy.fantuz@gmail.com (M.R.F.);

**Keywords:** recurrent implantation failure, chronic endometritis, gut microbiota, endometrial microbiota, intestinal dysbiosis, Rome IV, *Escherichia coli* Nissle 1917, IVF, Enterobacteriaceae, mucosal medicine

## Abstract

**Background**: Recurrent implantation failure (RIF) remains one of the most challenging conditions in reproductive medicine. Although increasing evidence implicates chronic endometritis (CE) and alterations of the endometrial microbiota in impaired implantation, the potential contribution of gastrointestinal dysfunction and intestinal dysbiosis remains poorly characterized. We hypothesized that, in a subgroup of women with pure RIF, reproductive failure may be associated with a broader mucosal phenotype involving both intestinal and endometrial compartments. **Objective**: To characterize the coexistence of gastrointestinal disorders, intestinal dysbiosis, CE, and endometrial microbial alterations in women diagnosed with pure RIF, and to descriptively report reproductive outcomes observed during subsequent multidisciplinary clinical management. **Methods**: This retrospective, single-centre, observational, hypothesis-generating study included women who met the strict ESHRE criteria for pure RIF, and were assessed at the “Federico II” IVF Centre, Naples, Italy. Patients underwent multidisciplinary assessment combining reproductive medicine, outpatient hysteroscopy, gastroenterological evaluation according to Rome IV criteria, conventional microbiological investigation and paired intestinal and endometrial microbiome characterization by 16S rRNA sequencing. Following clinical assessment, individualized gastroenterological management was undertaken according to routine practice. Reproductive outcomes were descriptively recorded during follow-up. **Results**: 19 women were analysed, all of whom met the Rome IV guidelines, and showed hysteroscopic and histologic signs of CE. Gut dysbiosis was identified in 94.7% (18/19) of patients, whereas endometrial microbial alterations were observed in 78.9% (15/19). In 84.2% (16/19) of the cohort, microbial profiles characterized by an abundance of Enterobacteriaceae in the intestinal and/or endometrial compartments were detected. Conventional microbiological positivity was present in less than half of patients, highlighting the distinction between microbiological infection and ecological microbial imbalance. During the follow-up period following completion of the personalized multidisciplinary treatment program, which included the administration of a well-characterized probiotic strain, *Escherichia coli* Nissle 1917, 11 women (57.9%) achieved a clinical pregnancy. Dysbiosis of the intestine was reported in 94.7% (18 out of 19) of participants, whereas endometrial microbiota disturbances were present in 78.9% (15 out of 19) of subjects. The Enterobacteriaceae-enriched microbiota profiles affecting the intestine and/or endometrium were seen in 84.2% (16 out of 19) of subjects. Out of 19 patients, 11 (57.9%) conceived during the follow-up period after gut-targeted treatment comprising *Escherichia coli* Nissle 1917. **Conclusions**: Women who meet strict criteria for pure RIF may represent a clinically identifiable subgroup characterized by the coexistence of gastrointestinal disorders, intestinal dysbiosis, CE, and endometrial microbial alterations. Rather than demonstrating therapeutic efficacy, the present study provides a translational and hypothesis-generating framework supporting the need for prospective validation of this integrated biological phenotype in women with pure RIF before evaluating personalized multidisciplinary management. Prospective controlled studies are warranted to evaluate whether gut-directed interventions may influence reproductive outcomes in this population.

## 1. Introduction

Recurrent implantation failure (RIF) remains one of the most challenging and controversial conditions in contemporary reproductive medicine, representing a major clinical, emotional, socio-economic burden for affected couples.

Despite remarkable advances in assisted reproductive technologies (ART), including optimization of ovarian stimulation protocols, embryo culture systems, blastocyst transfer, preimplantation genetic testing and embryo selection, a subgroup of women continues to experience RIF, and despite apparently favourable embryological and uterine conditions.

According to the most recent recommendations of the European Society of Human Reproduction and Embryology (ESHRE), RIF should no longer be considered a single disease entity but rather a heterogeneous clinical condition resulting from the complex interaction of embryonic, endometrial, immunological, endocrine, vascular and microbial determinants [1,2,3]. Consequently, RIF should be regarded as the final clinical manifestation of multiple biological mechanisms rather than the consequence of a single pathological process. This paradigm shift has progressively redirected research from the investigation of isolated reproductive abnormalities towards the identification of biologically meaningful patient subgroups sharing common pathophysiological mechanisms. Such an approach is expected not only to improve our understanding of implantation biology but also to promote the development of more personalized diagnostic pathways and physiopathology-driven therapeutic strategies for women who continue to experience implantation failure despite optimal reproductive management.

Successful embryo implantation represents one of the most sophisticated biological processes in human reproduction [4,5], requiring precise synchronization between embryonic competence and endometrial receptivity together with finely regulated immune tolerance, epithelial integrity, angiogenesis, extracellular matrix remodeling and metabolic homeostasis.

**Figure 1:**
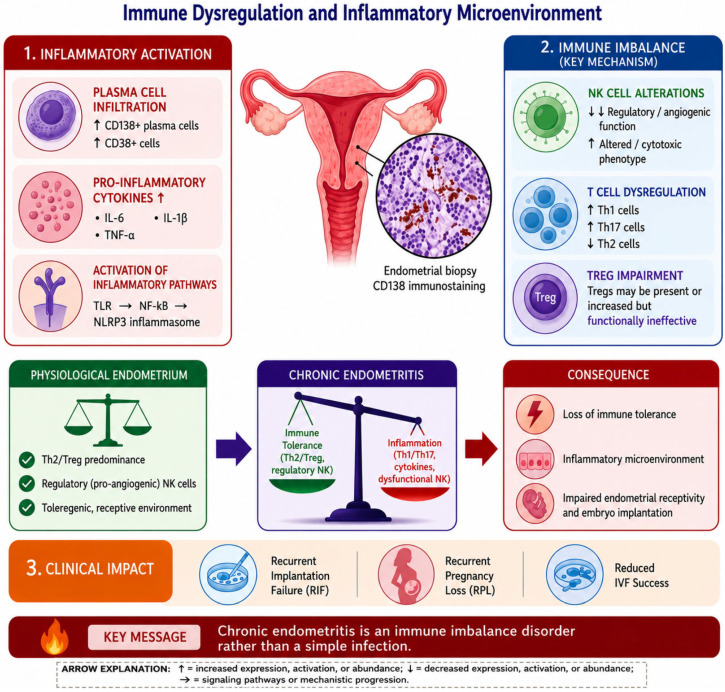
Schematic illustration of the immunological basis for chronic endometritis. Factors such as the presence of plasma cells (CD138 positive), up-regulated cytokines, and inflammation-related signalling pathway activation help create an altered state of endometrial immune balance. The altered state involves a dysfunction of NK cells, alteration in T cells, and decreased regulatory T cells (Tregs). This leads to the transition from immune tolerance to chronic inflammation. Such inflammation leads to poor endometrial receptivity, RIF, RPL, and poor outcomes in the use of assisted reproduction techniques. These results provide evidence for chronic endometritis being a disease involving immune dysregulation.

Even subtle disturbances affecting one or more of these interconnected mechanisms may compromise implantation without producing overt anatomical abnormalities. Consequently, increasing efforts have been devoted to identifying biological factors capable of influencing endometrial receptivity beyond the conventional evaluation of uterine anatomy and embryo quality. Among these, the reproductive microbiome has emerged as one of the most rapidly evolving areas of investigation. Several studies have demonstrated that alterations in the vaginal and endometrial microbial communities, particularly reduced Lactobacillus dominance and increased bacterial diversity may be associated with impaired implantation, RIF and adverse pregnancy outcomes [6,7,8]. Likewise, CE characterized by persistent low-grade endometrial inflammation, plasma cell infiltration, and a complex local immune-dysregulation, as represented in Figure 1, has consistently emerged as one of the most prevalent endometrial abnormalities identified in women with RIF [9]. Collectively, these observations have substantially expanded our understanding of the local endometrial microenvironment. Nevertheless, they continue to investigate the reproductive tract predominantly as an isolated biological compartment and therefore provide only a partial explanation for the complex pathophysiology underlying implantation failure in a substantial proportion of patients.

Recent advances in mucosal immunology and microbial ecology have progressively challenged the traditional organ-centred interpretation of female reproductive disorders. Rather than functioning as isolated anatomical compartments, mucosal tissues constitute an integrated biological network continuously communicating through immune signalling, microbial metabolites and epithelial barrier regulation. Within this network, the gastrointestinal tract represents the largest microbial and immunological interface of the human body and plays a fundamental role in maintaining systemic immune homeostasis. Beyond its digestive function, the intestinal microbiota actively contributes to epithelial barrier integrity, short-chain fatty acid production, bile acid metabolism, estrogen metabolism, regulatory T-cell differentiation and modulation of both innate and adaptive immune responses. Consequently, disturbances affecting intestinal microbial ecology may extend beyond the gastrointestinal tract, influencing distant organs through interconnected inflammatory, metabolic and endocrine pathways [10,11].

Within this evolving biological framework, the hypothesis of a gut–endometrial axis has progressively emerged. According to this hypothesis, intestinal dysbiosis may contribute to alterations in epithelial barrier function, persistent low-grade systemic inflammation and immune dysregulation, thereby creating biological conditions capable of influencing endometrial receptivity. Importantly, this model does not imply direct microbial translocation from the intestine to the endometrium but rather proposes that communication between these compartments occurs through complex immunological and metabolic pathways regulating mucosal homeostasis. Such a perspective is consistent with the growing recognition that several chronic inflammatory diseases involve coordinated alterations of multiple mucosal surfaces rather than isolated organ-specific abnormalities.

Although accumulating experimental and translational evidence supports the biological plausibility of this concept, its clinical relevance in reproductive medicine remains largely unexplored. Most available studies have investigated either the vaginal microbiome, the endometrial microbiome or chronic endometritis separately, whereas comprehensive clinical studies simultaneously integrating gastrointestinal symptoms, intestinal dysbiosis, chronic endometritis and endometrial microbial alterations remain remarkably scarce. Consequently, whether these abnormalities coexist as part of a reproducible clinical phenotype in women with pure RIF remains an unanswered question deserving further investigation.

Routine multidisciplinary clinical practice at our public IVF centre progressively drew our attention to a recurring observation. Women fulfilling strict contemporary ESHRE criteria for pure RIF frequently presented a remarkably homogeneous clinical profile extending well beyond reproductive failure itself. Alongside repeated implantation failure, these patients commonly reported gastrointestinal symptoms fulfilling Rome IV diagnostic criteria, predominantly severe functional constipation or constipation-predominant irritable bowel syndrome, demonstrated evidence of intestinal dysbiosis, exhibited hysteroscopic findings consistent with CE, and showed alterations involving the endometrial microbiota. Rather than occurring independently, these abnormalities repeatedly clustered within the same highly selected patients, suggesting the existence of a reproducible clinical phenotype characterized by coordinated disturbances affecting both intestinal and endometrial mucosal compartments.

This observation progressively modified our clinical approach. Instead of considering gastrointestinal symptoms, intestinal dysbiosis, CE and endometrial microbial alterations as unrelated findings, managed independently by different specialists, patients underwent an integrated multidisciplinary evaluation combining reproductive medicine, office hysteroscopy, gastroenterological assessment and paired gut–endometrial microbiome characterization.

The purpose of this approach was not to investigate each abnormality in isolation but to achieve a comprehensive clinical characterization capable of identifying shared biological patterns potentially underlying RIF.

Importantly, the present study did not originate from the intention to evaluate the efficacy of a specific probiotic intervention. Rather, it arose from the recognition of a reproducible clinical phenotype that generated a physiopathology-driven diagnostic and therapeutic pathway.

## 2. Materials and Methods

### 2.1. Study Design

This retrospective, single-centre, observational, hypothesis-generating study, was conducted at the Department of Maternal and Child Health, University Federico II of Naples, Italy, between January and December 2025. The study originated from recurring clinical observations made during the routine multidisciplinary management of women fulfilling contemporary ESHRE criteria for pure RIF.

Clinical, hysteroscopic, microbiological, microbiome and reproductive data generated during routine patient care were retrospectively collected and analysed. No additional diagnostic or therapeutic procedures were introduced for research purposes, and patient management was not influenced by study participation.

The study was conducted and reported in accordance with the Strengthening the Reporting of Observational Studies in Epidemiology (STROBE) statement for observational studies.

All consecutive women attending the University Federico II IVF Centre during the study period were screened for eligibility. Women aged ≤38 years who fulfilled the contemporary ESHRE diagnostic criteria for pure RIF were considered eligible. Patients with endometriosis, untreated hydrosalpinx, congenital uterine anomalies, parental chromosomal abnormalities, uncontrolled endocrine disorders, autoimmune diseases or inflammatory bowel diseases were excluded, in order to minimize potential confounding factors affecting implantation.

During the study period, twenty-five women fulfilled all predefined eligibility criteria and entered the multidisciplinary diagnostic pathway. At the time of data collection, nineteen women had completed both the diagnostic evaluation and reproductive follow-up and therefore constituted the final study population included in the present analysis. Six women had not yet reached reproductive outcome assessment at the time of analysis.

All eligible patients underwent a standardized multidisciplinary diagnostic assessment integrating reproductive medicine, office hysteroscopy, gastroenterological evaluation, conventional microbiological investigation and paired intestinal and endometrial microbiome characterization.

Accordingly, each patient was evaluated through an integrated multidisciplinary team, which included reproductive medicine specialists, experienced hysteroscopists and a gastroenterologist with expertise in disorders of gut–brain interaction and intestinal microbiota.

Hysteroscopic procedures were performed in an outpatient setting [12], using a vaginoscopic approach with a 5 mm Bettocchi continuous flow operating hysteroscope (Karl Storz, Germany), according to routine clinical practice. Hysteroscopic findings indicative of CE were recorded, including focal or diffuse hyperaemia, stromal oedema and micropolyps; the co-occurrence of these three hysteroscopic signs has, in fact, a proven diagnostic accuracy of 93.4% [13]. A targeted endometrial biopsy was performed for histopathological confirmation via immunohistochemistry with CD138, using a 5 Fr “grasping” forceps.

A standardized gastroenterological evaluation was performed by the same gastroenterologist (F.C.) to ensure methodological consistency throughout the study. Gastrointestinal disorders were classified according to the Rome IV Diagnostic Criteria, the current international reference standard for Disorders of Gut–Brain Interaction (DGBI).

The clinical assessment included a comprehensive evaluation of bowel habits, stool consistency, abdominal symptoms, dietary habits, previous gastrointestinal diagnoses, prior microbiota-oriented treatments, antibiotic exposure and symptom duration. Based on the predominant clinical presentation, patients were classified as having severe functional constipation, constipation-predominant irritable bowel syndrome (IBS-C), diarrhoea-predominant irritable bowel syndrome (IBS-D) or mixed irritable bowel syndrome (IBS-M), according to Rome IV definitions.

Conventional cervical and/or endometrial cultures were performed whenever clinically indicated according to routine gynaecological practice. Microbiological investigations were requested on the basis of clinical history, previous reproductive investigations and hysteroscopic findings to identify cultivable microorganisms potentially associated with active genital tract infection.

Paired faecal and endometrial samples were collected before the initiation of the individualized gastroenterological management. The endometrial sample was obtained by hysteroscopically guided endometrial biopsy, using 5 Fr “grasping” forceps. Both specimens were analysed by next-generation sequencing (NGS) targeting the bacterial 16S rRNA gene, according to the standardized analytical workflow of the reference laboratory.

Particular attention was devoted to the relative abundance of *Lactobacillus* spp. within the endometrial microbiota and Enterobacteriaceae within both intestinal and endometrial compartments because of their potential biological relevance to the hypothesis of an integrated gut–endometrial phenotype. Because no universally accepted international criteria currently exist for the diagnosis of either intestinal or endometrial dysbiosis, microbiome interpretation and therapeutic decisions were based on the comprehensive ecological assessment provided by the reference laboratory and integrated with clinical, hysteroscopic and conventional microbiological findings.

Following completion of the multidisciplinary diagnostic assessment, patients underwent individualized gastroenterological management according to routine clinical practice. Treatment was tailored to each patient’s Rome IV diagnosis, gastrointestinal phenotype, microbiome profile and overall clinical assessment.

All patients received *Escherichia coli* Nissle 1917 (ECN) at a dose of 5 × 10^9^ CFU/day (2 capsules/24 h) for 12 weeks, as part of an individualized, multidisciplinary, physiopathology-driven management strategy. Additional dietary or lifestyle recommendations were provided when clinically indicated according to routine gastroenterological practice.

*ECN* was selected because of its established immunomodulatory properties, documented effects on epithelial barrier function and long-standing clinical use in gastroenterology, particularly for maintenance of remission in ulcerative colitis. Therefore, its use reflected a biologically plausible gastroenterological strategy consistent with the observed clinical phenotype rather than an intervention specifically designed to improve reproductive outcomes.

### 2.2. Statistical Analysis

Statistical analyses were performed using IBM SPSS Statistics version 30.0.0.0 (IBM Corp., Armonk, NY, USA). Given the exploratory and hypothesis-generating nature of the study, together with the limited sample size, the statistical analysis was intentionally restricted to descriptive statistics.

Continuous variables are presented as mean ± standard deviation (SD) or median with interquartile range (IQR), according to data distribution, whereas categorical variables are expressed as absolute numbers and percentages.

No inferential statistical analyses were performed to evaluate treatment efficacy, as the study was neither designed nor powered to assess therapeutic effectiveness. Accordingly, reproductive outcomes are reported descriptively and should be interpreted exclusively within the context of this retrospective observational study.

### 2.3. Ethics

This study retrospectively analysed anonymized clinical data generated during routine multidisciplinary patient care. No additional diagnostic or therapeutic procedures were performed specifically for research purposes, and patient management was not modified by study participation.

All patients had previously provided written informed consent for diagnostic procedures and for the anonymous use of their clinical data for research purposes.

The study was conducted in accordance with the ethical principles of the Declaration of Helsinki and complied with the institutional ethical requirements of the University Federico II of Naples.

## 3. Results

Nineteen women fulfilled all predefined eligibility criteria, completed both the multidisciplinary diagnostic pathway and reproductive follow-up, and constituted the final study population. Baseline demographic characteristics are reported in Table 1, while gastrointestinal and reproductive characteristics are summarized in Table 2.

Mean age was 34.2 ± 3.5 years, and the mean body mass index (BMI) was 27.8 ± 2.9 kg/m^2^.

The cohort had a history of repeated embryo transfer failures, with a median (IQR) of 4.5 (3.75–6.00) previous failed embryo transfers. A total of 56/74 (75.67%) previous transfers were frozen embryo transfers (FET), whereas 18/74 (24.32%) were fresh transfers; all patients enrolled underwent transfer of one embryo. Smoking was reported by 3/19 (15.8%) of patients, while occasional (i.e., non-habitual) alcohol consumption was reported in all patients.

Owning to the predefined inclusion and exclusion criteria, the cohort represented a clinically homogeneous population of women fulfilling the contemporary ESHRE definition of pure RIF.

All enrolled women fulfilled the Rome IV diagnostic criteria for DGBI. Severe functional constipation represented the predominant clinical phenotype, affecting 57.9% (11/19) of patients, followed by IBS-C in 21.1% (4/19), IBS-D in 15.8% (3/19) and IBS-M in 5.3% (1/19).

Hysteroscopic criteria for diagnosis of CE, and histological confirmation through CD138 immunohistochemistry, were detected in 19/19 women (100%).

Conventional microbiological cultures were positive in 47.4% (9/19) of patients. Microbiome analysis demonstrated intestinal dysbiosis in 94.7% (18/19) of patients and endometrial microbial alterations in 78.9% (15/19).

Enterobacteriaceae-enriched microbial profiles involving the intestinal and/or endometrial compartment were identified in 84.2% (16/19) of the cohort. Detailed microbiome findings are reported in Table 2.

During reproductive follow-up, 11 of 19 women (57.9%) achieved a clinical pregnancy. Among these, two resulted in early miscarriage, two in term live birth, and seven were ongoing at the time of data collection. Accordingly, the ongoing pregnancy rate was 36.8% (7/19) and the live birth rate available at the time of analysis was 10.5% (2/19). Embryo-transfers performed and reproductive outcomes are reported in Table 3.

Given the observational design of the study and the absence of a comparison group, reproductive outcomes are presented descriptively and should not be interpreted as evidence of treatment efficacy.

## 4. Discussion

The present study suggests that women with pure RIF may share a remarkably homogeneous mucosal phenotype characterized by gastrointestinal dysfunction, intestinal dysbiosis, CE, and endometrial microbial alterations. Traditionally, RIF has been approached as a uterine and multifactorial disorder [14]. Our findings suggest a broader perspective in which implantation failure may represent the reproductive consequence of systemic mucosal dysregulation. The coexistence of intestinal and endometrial abnormalities is consistent with the emerging concept of a gut–endometrial axis mediated by immune, epithelial, and metabolic signalling pathways. Our data support the view that CE may represent a chronic inflammatory phenotype rather than a simple infection. The incomplete overlap between CE, microbiological positivity, and dysbiosis reinforces this interpretation.

An additional finding deserving consideration is that fewer than half of women with endometrial microbial alterations had positive conventional microbiological cultures. This discrepancy highlights the fundamental difference between microbiological infection and ecological dysbiosis. Conventional culture-based techniques identify cultivable microorganisms but provide limited information regarding the overall composition and ecological balance of the microbial community. In contrast, NGS detects shifts in microbial diversity and relative abundance that may occur even in the absence of cultivable pathogens. Therefore, a negative endometrial culture should not necessarily be interpreted as evidence of a physiologically balanced endometrial microbiota. These findings suggest that conventional microbiology and microbiome analysis provide complementary rather than interchangeable information and support the integration of both approaches in future studies investigating CE and RIF.

The observation that nearly half of these highly selected women subsequently achieved clinical pregnancy is clinically meaningful. Although causality cannot be inferred from the present retrospective analysis, the observed reproductive outcomes support further investigation of microbiota-oriented strategies in selected patients with RIF.

The present study was not designed to evaluate the therapeutic efficacy of ECN. Rather, its use was based on the gastrointestinal phenotype identified during multidisciplinary assessment and on its well-established biological properties.

Experimental and clinical studies have demonstrated that ECN may contribute to the restoration of mucosal immune homeostasis through modulation of epithelial barrier integrity, attenuation of NF-κB-dependent inflammatory signalling, and regulation of both innate and adaptive immune responses [15,16]. These mechanisms have been extensively investigated in gastroenterology, particularly in inflammatory bowel disease, where ECN has shown efficacy in maintaining remission of ulcerative colitis [17,18]. These mechanisms are particularly relevant considering the emerging concept that chronic endometritis represents a disorder of persistent mucosal immune dysregulation rather than merely a chronic infectious condition. Although no evidence currently supports a direct reproductive effect, these immunomodulatory and barrier-regulating properties provide a biologically plausible rationale for investigating gut-directed interventions in women presenting with the combined intestinal and endometrial phenotype observed in the present study.

Whether modulation of the intestinal ecosystem can influence endometrial inflammation and reproductive outcomes remains unknown and should be investigated in adequately powered prospective controlled studies [19,20].

Our findings should be interpreted within the emerging concept of the gut–endometrial axis. Although the mechanisms linking intestinal microbial ecology and endometrial homeostasis remain incompletely understood, increasing evidence suggests that disturbances in mucosal immune regulation may extend beyond individual organs and involve coordinated alterations of multiple mucosal compartments. The coexistence of gastrointestinal disorders, intestinal dysbiosis, CE and endometrial microbial alterations observed in our cohort supports the need for further investigation of this integrated biological phenotype in women with RIF.

### Strengths, Limitations and Future Perspectives

The present study should be interpreted within the context of its exploratory and hypothesis-generating design. Several limitations deserve consideration. The retrospective single-centre nature of the study, the relatively small sample size and the absence of an untreated comparison group preclude any inference regarding causality or treatment efficacy. Importantly, the absence of a matched control cohort reflects the original exploratory design of the study rather than a methodological omission. The present work was conceived as a descriptive observational cohort aimed at biological characterization of a highly selected clinical phenotype, whereas prospective controlled studies will be required to evaluate the therapeutic implication of this observation.

Similarly, the lack of longitudinal microbiome analyses does not allow assessment of temporal changes in microbial ecology following multidisciplinary management or the identification of the biological mechanisms potentially underlying the reproductive outcomes observed during follow-up.

Furthermore, severe male factor infertility was not considered an exclusion criterion. Although only one couple in our cohort was affected and subsequently achieved a viable pregnancy, the potential contribution of male infertility to reproductive outcomes cannot be completely excluded. Therefore, this factor should be considered a possible source of confounding and addressed through predefined eligibility criteria or stratified analyses in future prospective studies.

Nevertheless, these limitations should be interpreted considering the distinctive characteristics of the study population. Although only nineteen women were included in the final analysis, they represented the entire cohort of patients fulfilling the predefined eligibility criteria who presented to our tertiary referral IVF centre during the study period and had reached reproductive outcome assessment at the time of data analysis. The study was conducted at a high-volume tertiary referral IVF centre, where the stringent inclusion criteria were intentionally designed to identify a biologically homogeneous subgroup of women fulfilling contemporary ESHRE criteria for pure RIF. Therefore, the relatively small sample size reflects the rarity of this highly selected clinical phenotype rather than limited patient recruitment.

Another important strength of the study lies in its multidisciplinary and translational design, which allowed the comprehensive characterization of a reproducible clinical phenotype observed in routine practice. This bedside-to-bench approach led to the identification of a remarkably consistent clinical phenotype characterized by the coexistence of disorders of gut–brain interaction, CE, intestinal dysbiosis and endometrial microbial alterations. Although the mechanisms linking these findings remain to be elucidated, their reproducible coexistence provides a strong biological rationale for future mechanistic investigation. Future research should therefore move beyond the evaluation of isolated therapeutic interventions and focus on validating this integrated gut–endometrial phenotype through comprehensive biological characterization. Prospective multicentre studies integrating longitudinal microbiome profiling and controlled multidisciplinary interventions will be essential to determine whether this phenotype represents a distinct biological entity and whether its recognition may contribute to a more personalized approach to RIF.

## 5. Conclusions

In this retrospective observational study of all women with pure RIF evaluated in a public IVF centre, we identified a highly consistent phenotype characterized by Rome IV-positive gastrointestinal symptoms, intestinal dysbiosis, CE, and endometrial microbial alterations. These findings support the concept that, in a subset of patients, RIF may represent the reproductive expression of a broader mucosal inflammatory disorder involving the gut–endometrial axis. The observation that nearly half of the cohort subsequently achieved pregnancy during follow-up further highlights the potential clinical relevance of this model. Our results should be considered hypothesis-generating and provide a strong rationale for prospective translational studies aimed at redefining the pathophysiology and management of RIF.

## Figures and Tables

**Table 1:** Baseline Characteristics of the Study Population.

Variable	Total Cohort (n = 19)
Age (years), mean ± SD	34.3 ± 4.55
BMI (kg/m^2^), mean ± SD	24.06 ± 3.43
Patient current smokers, n (%)	3/19 (15.8%)
Occasional alcohol consumption, n (%)	19/19 (100%)
Duration of infertility (years), mean ± SD	6.53 ± 1.80
Previous ART cycles, mean ± SD	2.93 ± 1.38
Homologous ART cycles, n (%)	19/19 (100%)
Donor ART cycles, n (%)	0/19 (0%)
Previous Failed Fresh ET, n (%)	18/74 (24.32%)
Previous FET, n (%)	56/74 (75.67%)
Previous Failed Fresh ET, mean ± SD	1.2 ± 1.85
Previous FET, mean ± SD	3.73 ± 2.15
Previous failed ET, mean ± SD	2.46 ± 2.35

SD: Standard Deviation, BMI: Body Mass Index, ART: Assisted Reproductive Technology, ET: Embryo Transfer, FET: Frozen Embryo Transfer.

**Table 2:** Gastrointestinal and Endometrial Characteristics of the Study Population.

Variable	Total Cohort (n = 19)
Severe constipation, n (%)	11/19 (57.9%)
IBS-C, n (%)	4/19 (21.1%)
IBS-D, n (%)	3/19 (15.8%)
IBS-M, n (%)	1/19 (5.3%)
Intestinal dysbiosis, n (%)	18/19 (94.7%)
Chronic endometritis, n (%)	19/19 (100%)
Endometrial dysbiosis, n (%)	15/19 (78.9%)
Enterobacteriaceae enrichment, n (%)	16/19 (84.2%)

IBS-C: constipation-predominant irritable bowel syndrome, IBS-D: diarrhoea-predominant irritable bowel syndrome, IBS-M: mixed irritable bowel syndrome.

**Table 3:** Reproductive Outcomes Following Gut-Directed Therapy Including *Escherichia coli* Nissle 1917.

Outcome	Total Cohort (n = 19)
FET, n (%)	17/19 (89.47%)
Fresh ET, n (%)	2/19 (10.52%)
Pregnancy achieved, n (%)	11/19 (57.9%)
Miscarriage, n (%)	2/19 (10.5%)
Ongoing pregnancy, n (%)	7/19 (36.8%)
Live birth, n (%)	2/19 (10.5%)

FET: Frozen Embryo Transfer; ET: Embryo Transfer.

## Data Availability

The data presented in this study are available on request from the corresponding author due to privacy.
